# Ausfall kritischer Infrastrukturen in deutschen Krankenhäusern: Onlineumfrage zu Krisenstabsstrukturen und Übungen in Krankenhäusern

**DOI:** 10.1007/s00101-026-01664-4

**Published:** 2026-03-27

**Authors:** Manuel Geiger, Steffen Neuner, Muhammed Enes Bodur, Alexander Fekete

**Affiliations:** Institut für Rettungsingenieurwesen und Gefahrenabwehr, TH Köln, 50679 Köln, Deutschland

**Keywords:** Krankenhausalarm- und -einsatzplanung, KAEP, Krisenmanagement, Krankenhauseinsatzleitung, Notfallplanung, Hospital incident action planning, Crisis management, Hospital incident management team, Emergency planning, Supply

## Abstract

**Hintergrund:**

Krankenhäuser sind kritische Infrastrukturen, die selbst auf versorgende kritische Infrastrukturen angewiesen sind. Für die Reaktion auf unerwartete Ereignisse sind neben technischen Aspekten auch organisatorische Strukturen notwendig.

**Ziel:**

Die Studie hat das Ziel, die eingesetzten Stabsstrukturen und die Häufigkeit von Übungen, insbesondere zu Ausfällen von kritischen Infrastrukturen, zu ermitteln.

**Methoden:**

Eine Onlineumfrage wurde von November 2022 bis April 2023 durchgeführt und insgesamt 72-mal vollständig ausgefüllt.

**Ergebnisse:**

Die Ergebnisse zeigen, dass 47 % der Krankenhäuser die vom Bundesamt für Bevölkerungsschutz und Katastrophenhilfe empfohlenen Stabsstrukturen der Feuerwehr-Dienstvorschrift 100 nutzen. Speziell in Krankenhäusern mit weniger als 200 Betten ist die Heterogenität erhöht, und es wird häufiger in anderen Strukturen gearbeitet. Übungshäufigkeit und -arten variieren stark: Es führen 67 % der Krankenhäuser nur selten (einmalig letzte 5 Jahre) oder nie Übungen durch, während größere Krankenhäuser häufiger Übungen abhalten. Besonders Übungen zu Stromausfällen werden regelmäßig durchgeführt (ca. 53 % mindestens jährlich), während Übungen zu Trinkwasserversorgung und Abwasserentsorgung selten sind. Es wird ein Trend erkennbar, dass größere Krankenhäuser mit mehr Betten häufiger üben und auch komplexere Übungen (z. B. Realübungen) durchführen.

**Diskussion:**

Die Diskrepanz in der Übungsfrequenz zwischen großen und kleinen Krankenhäusern lässt vermuten, dass die Durchführung von Übungen eine Herausforderung für kleinere Krankenhäuser darstellt. Es wird empfohlen, angepasste Stabsstrukturen zu entwickeln und Übungen stärker in den Fokus zu rücken, besonders im Bereich kritischer Infrastrukturausfälle wie Trinkwasserversorgung und Abwasserentsorgung.

**Zusatzmaterial online:**

Zusätzliche Informationen sind in der Online-Version dieses Artikels (10.1007/s00101-026-01664-4) enthalten.

## Abstract (en)

**Background:**

Hospitals are critical infrastructures and essential for healthcare provision, yet they heavily depend on the continuous availability of other parts of the critical infrastructure, such as electricity and water supply. In emergencies, organizational aspects often play a crucial role as hospitals need to shift from routine organizational structures to specialized crisis management frameworks. This survey investigates the organizational structures used by German hospitals in crisis management, focusing on the frequency and types of exercises related to critical infrastructure failures, particularly water and power supply as well as sewage disposal.

**Objective:**

While organizational preparations for mass casualty incidents are common in German hospitals, insufficient structures are implemented for critical infrastructure failures as well as how often these scenarios are practiced. This study aims to address these gaps by examining hospital crisis management structures, the frequency of related exercises and the interplay among hospital size, ownership and regional factors. The research is part of the NOWATER project, funded by the Federal Ministry of Education and Research (BMBF, 13N15281), which investigates these aspects.

**Methods:**

The survey conducted from November 2022 to April 2023 and targeting hospitals across Germany, gathered 72 fully completed responses, representing 3.8% of Germanyʼs total hospitals.

**Results:**

The findings reveal a diverse range of crisis management structures employed across hospitals, with 47% adhering to the federal recommendation for the *Feuerwehr-Dienstvorschrift 100* (Fire Department service regulations 100) framework; however, many hospitals, especially those with fewer than 200 beds, opt for regular organizational structures or hybrid models. The survey also highlights a significant variation in the frequency of exercises across hospitals. While annual exercises for power failure scenarios are common, training related to water and wastewater disruptions is rarely conducted. Additionally, hospitals with fewer than 200 beds tend to conduct fewer exercises than larger hospitals, with significant disparities in resources influencing exercise frequency and type.

**Conclusion:**

The results suggest that while larger hospitals are more likely to follow recommended crisis management structures and conduct regular exercises, smaller hospitals often lack the personnel and financial resources necessary for such preparations. This disparity underlines the need for tailored crisis management strategies that account for hospital size and available resources. In conclusion, the study underlines the importance of regular crisis management exercises to improve resilience and preparedness for infrastructure failures. It also calls for increased focus on water and wastewater contingency planning, which currently lags behind emergency power supply preparations in most hospitals. Policymakers and healthcare managers are encouraged to support hospitals, particularly smaller ones, with the necessary resources to implement effective crisis management structures and exercises.

## Hinführung zum Thema

Krankenhäuser sind kritische Infrastrukturen und für die Gesundheitsversorgung unabdingbar. Gleichzeitig sind sie selbst von einer dauerhaften Verfügbarkeit versorgender kritischer Infrastrukturen, wie der Strom- und Trinkwasserversorgung, abhängig [1]. Jedoch spielen nicht nur technische Aspekte eine wichtige Rolle, speziell bei unerwarteten Ereignissen haben die organisatorischen Aspekte einen entscheidenden Einfluss. Die Bewältigung von Ereignissen hängt von der Fähigkeit eines Krankenhauses ab, schnell von üblichen organisatorischen Strukturen auf besondere Organisationsstrukturen umzuschalten.

## Hintergrund

Organisatorische Vorbereitungen auf außergewöhnliche Ereignisse variieren je nach potenziellem Ereignis. Während Vorbereitungen für einen Massenanfall von Verletzten (MANV) in deutschen Krankenhäusern etabliert und wissenschaftlich untersucht sind [2–7], fehlt es an Arbeiten zum Stand der Vorbereitung auf den Ausfall versorgender kritischer Infrastrukturen. Die organisatorischen Vorbereitungen auf außergewöhnliche Ereignisse umfassen u. a. die Einrichtung und Übung von Krisenstäben [6, 8–11]. Zu den hierfür notwendigen Stabstrukturen gibt es eine Empfehlung des Bundesamts für Bevölkerungsschutz und Katastrophenhilfe (BBK) [12], die eine leicht modifizierte Version der Feuerwehr-Dienstvorschrift 100 „Führung und Leitung im Einsatz“ (FwDV 100) [13] darstellt. Diese umfasst alle Sachgebiete S1–S6 in der Annahme, dass die Zusammenarbeit von Behörden und Organisationen mit Sicherheitsaufgaben (BOS) und Krankenhäusern durch die funktions- und organisationsgleichen Stabsstrukturen verbessert wird. Eine individuelle Anpassung der Stabsstrukturen an die Begebenheiten im eigenen Krankenhaus ist empfohlen. In welchem Umfang die empfohlenen Stabsstrukturen in Krankenhäusern zur Anwendung kommen, ist derzeit unzureichend bekannt. Ebenfalls ist unzureichend untersucht, wie oft diese Stabsstrukturen sowie Infrastrukturausfälle in Krankenhäusern geübt werden. Im Rahmen des Forschungsprojektes NOtfallvorsorgeplanung der WAsserver- und -entsorgung von Einrichtungen des Gesundheitswesens – organisatorische und Technische Lösungsstrategien zur Erhöhung der Resilienz (NOWATER), welches vom Bundesministerium für Bildung und Forschung unter dem Förderkennzeichen 13N15281 gefördert wurde, sind die aufgeführten Fragestellungen als Grundlage für die weiteren Arbeiten untersucht worden.

## Fragestellung

In der Umfrage sollen folgende Fragestellungen untersucht werden:

- Welche Stabsstrukturen werden in deutschen Krankenhäusern verwendet?
- Wie häufig werden in welcher Form in Krankenhäusern welche außergewöhnlichen Ereignisse geübt?
- Wie häufig wird der Ausfall von kritischen Infrastrukturen, insbesondere der Trinkwasser- und Stromversorgung sowie der Abwasserentsorgung, geübt?

## Methode der Onlinebefragung

Um mögliche Unterschiede auf die Organisation der Stabsarbeit in Krankenhäusern verschiedener Größe und Lagen untersuchen zu können, wurden über die Kernfragen hinausgehend die demografischen und strukturellen Daten der Krankenhäuser erhoben. Diese umfassen das Bundesland, die Trägerschaft, das Versorgungslevel und die Bettenzahl. Anschließend wurden die Untersuchungsinhalte (abhängige Variablen) wie verwendete Stabsstrukturen und Häufigkeiten, Themen und Arten von durchgeführten Übungen abgefragt. Der Fragebogen ist als Zusatzmaterial online abrufbar. Die Primärergebnisse stehen auf Anfrage in anonymisierter Form zur Verfügung.

Start der Befragung war am 16.11.2022 und Ende am 15.02.2023. Zur Erhöhung der Teilnahmen wurde die Umfrage erneut vom 01.03.2023 bis zum 15.04.2023 verteilt. Die Umfrage wurde nicht randomisiert, nicht selektiv, in einem explorativen Ansatz über die Landeskrankenhausgesellschaften, einen bundesweiten E‑Mail-Verteiler aller Krankenhäuser aus dem Krankenhausregister sowie unterschiedliche Fachgesellschaften aus dem Bereich Krankenhaustechnik verteilt.

Die Auswertung erfolgte in zwei Schritten. Erster Schritt war eine allgemeine Betrachtung der Ergebnisse zur Bewertung der Nutzbarkeit sowie die rein quantitative Analyse. Die Trägerschaft und die Größe des Krankenhauses (Bettenzahl, Versorgungslevel) wurden im Anschluss mit den verwendeten Stabsstrukturen sowie der Anzahl bzw. dem Intervall und der Art der Übungen verglichen.

## Ergebnis

### Gesamtzahlen der Umfrage und statistische Eingrenzungen

**Abb. 1:**
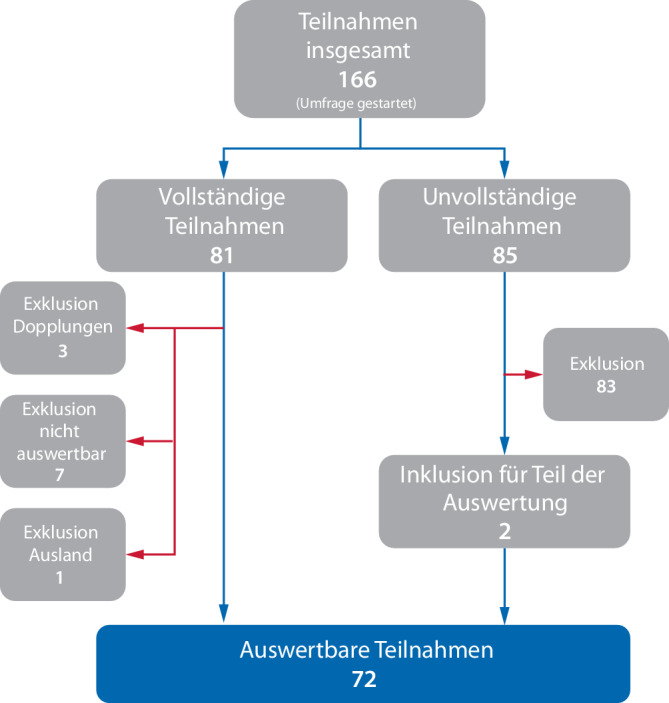
Nummerische Auswertung der Umfrage (eigene Darstellung)

Die Umfrage wurde von insgesamt 166 Personen begonnen und 85-mal vollständig abgeschlossen. Zudem wurden in die Auswertung 2 unvollständige Umfragen (Teilnahme bis zur Fragenmatrix zu Übungen in Krankenhaus) in die Auswertung aufgenommen, die fehlenden Antworten durch die Antwortmöglichkeit „Unbekannt“ ergänzt. Insgesamt mussten 11 vollständig abgeschlossene Umfragen ausgeschlossen werden (Abb. 1). Somit ergibt sich eine Summe von 72 vollständig ausgefüllten und für die weitere Auswertung verwertbaren Teilnahmen. Im Hinblick auf eine Krankenhauslandschaft von 1887 (Stand 2021) Krankenhäusern deutschlandweit, ergibt das eine Stichprobe von 3,8 % aller deutschen Krankenhäuser [14].

Aus den Bundesländern Niedersachsen und Schleswig-Holstein nahm kein Krankenhaus an der Umfrage teil. Zudem sind 50 von 72 Teilnahmen aus den Bundesländern Baden-Württemberg (16), Nordrhein-Westfalen (24) und Rheinland-Pfalz (10). Alle weiteren Bundesländer sind gleichmäßig mit einer bis vier Teilnahmen vertreten. Als erwartete Einflussfaktoren wurden die Trägerschaft, das Bundesland und die Größe der Krankenhäuser in Form der Bettenzahl abgefragt. Es zeigt sich, dass die Bettenzahl stärkster beeinflussender Faktor ist, weshalb diese für die weitere Auswertung als Kenngröße herangezogen wird.

### Vergleich der Krankenhäuser nach Bettenzahl

Der Vergleich der Stichprobe der Umfrage im Vergleich mit der deutschen Krankenhauslandschaft ist in Tab. 1 dargestellt. Diese zeigt, dass v. a. kleine Krankenhäuser mit weniger als 200 Betten in der Umfrage unterrepräsentiert sind. In der deutschen Krankenhauslandschaft bilden sie jedoch mit 56 % den größten Anteil [15]. Zudem sind große Krankenhäuser mit 600 Betten und mehr in der Umfrage deutlich überrepräsentiert.

**Tab. 1:** Vergleich der Bettenzahlen von Krankenhäusern in Deutschland [15] mit Stichprobe der Umfrage

Bettenzahl	Deutschland absolut	Deutschland prozentual (%)	Umfrage absolut	Umfrage prozentual (%)	Differenz Umfrage zu Krankenhauslandschaft (%)
*0–199*	1054	56	16	22	−33
*200–399*	432	23	21	30	+7
*400–599*	223	12	8	11	−1
*600–1000*	178	9	11	15	+25
*>* *1000*			15	21	
*Unbekannt*	–	–	1	1	–
*Gesamt*	*1887*	–	*72*	–	–

Eine Analyse der Krankenhauslandschaft, basierend auf öffentlich verfügbaren Daten, in Deutschland ergibt, dass von 1887 Krankenhäusern 72 % in privatwirtschaftlicher (*n* = 733) oder freigemeinnütziger (*n* = 607) Trägerschaft sind, und 28 % (*n* = 547) in öffentlicher Hand [14]. Rund 56 % (1054) dieser Krankenhäuser haben 0 bis 199 Betten, 432 haben 200 bis 399 Betten (ca. 23 %), 223 Krankenhäuser haben 400 bis 599 Betten (ca. 12 %), und 178 Krankenhäuser (ca. 9 %) haben 600 und mehr Betten [15]. Die Mehrzahl der Krankenhäuser verfügt also über weniger als 200 Betten [15].

### Verwendete Stabsstrukturen in Krankenhäusern

**Abb. 2:**
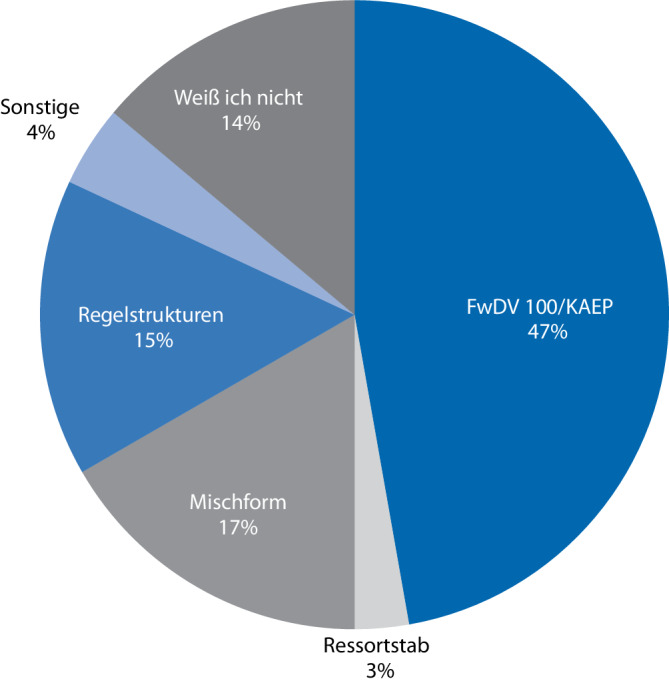
Vergleich der eingesetzten Stabsstrukturen in deutschen Krankenhäusern aus der durchgeführten Umfrage (*n* = 72) (eigene Darstellung)

Die häufig empfohlenen Strukturen der FwDV 100 [11, 12] werden von 47 % der Krankenhäuser eingesetzt. Während 3 % der Krankenhäuser einen Ressortstab ähnlich den Krisenstäben der öffentlichen Verwaltung nutzen, ziehen 17 % eine Mischform von Stabsstrukturen vor. Rund 15 % der Krankenhäuser arbeiten in den regulären Strukturen, und bei 4 % „Sonstige“ sind die Stabsstrukturen nicht näher definiert, und 14 % der Teilnehmenden kannten die Stabsstrukturen nicht. Die Ergebnisse sind in Abb. 2 grafisch dargestellt.

**Abb. 3:**
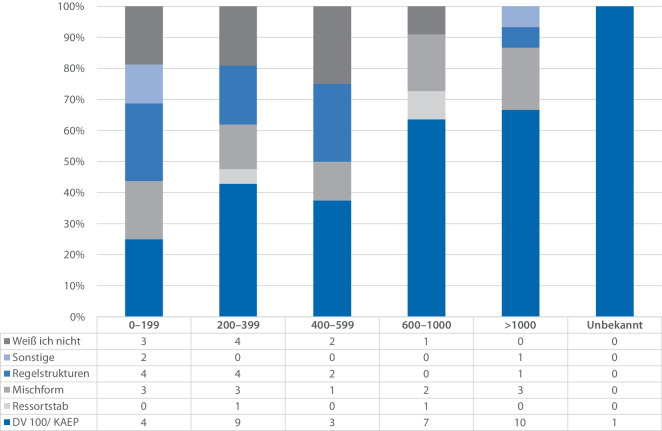
Vergleich der eingesetzten Stabsstrukturen nach Anzahl an Betten als Ergebnis der Umfrage (*n* = 72), jeweils relative Angaben zur Anzahl der Antworten in der Untergruppe. Ergänzend findet sich unter der Abbildung eine Tabelle mit den absoluten Zahlen der Umfrage (eigene Darstellung)

Um die verwendeten Stabsstrukturen und die anwendenden Krankenhäuser näher einzugrenzen, erfolgt im Weiteren die detaillierte Aufschlüsselung von Stabsstrukturen nach Bettenzahl der Krankenhäuser (Abb. 3) Es zeigt sich ein allgemein heterogenes Bild der angewendeten Stabsstrukturen.

Es ist erkennbar, dass v. a. kleinere Krankenhäuser mit bis zu 199 Betten verhältnismäßig seltener in den Strukturen der FwDV 100 bzw. den empfohlenen Strukturen der Krankenhausalarm- und -einsatzplanung (KAEP) arbeiten, als dies in großen Krankenhäusern der Fall ist. Ergänzend ist die Arbeit in Regelstrukturen in der Gruppe der kleineren Krankenhäuser genauso häufig wie die Arbeit in den Strukturen der FwDV 100. Vor allem in der Gruppe mit 200 bis 399 Betten, und in Krankenhäusern mit 600 und mehr Betten finden die Stabsstrukturen nach FwDV 100 vermehrt Anwendung. Zudem zeigt sich, dass in fast jeder Gruppe das Krisenmanagement in Regelstrukturen und nicht in Stabsstrukturen oder in Mischformen bearbeitet wird. Stäbe nach dem Ressortprinzip finden sich im Vergleich seltener. Allgemein zeigt sich ein sehr heterogenes Bild, sodass ein klarer Zusammenhang von Bettenzahl und Stabsstrukturen nicht erkennbar ist.

### Häufigkeit von Übungen der Krankenhauseinsatzleitung zum Ausfall kritischer Infrastruktur

Zur Untersuchung der Häufigkeit von Übungen mit dem Thema Ausfall kritischer Infrastrukturen in Krankenhäusern wurden die Anzahl der Übungen sowie die Thematik der Übungen in der Umfrage erhoben. Zudem wurde die Art der Übung mitabgefragt. Im Folgenden werden die Ergebnisse allgemein und detailliert nach Bettenzahl aufgeschlüsselt dargestellt.

**Abb. 4:**
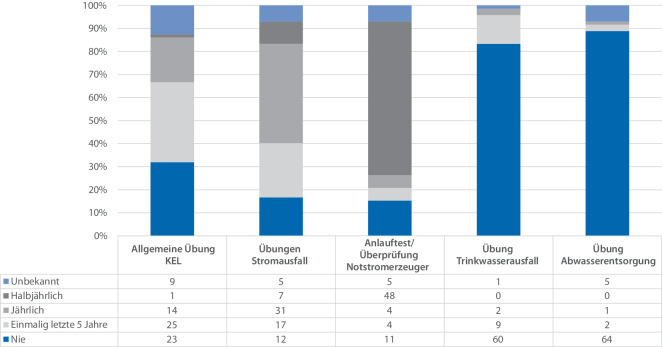
Auswertung der Häufigkeit von Übungen der Krankenhauseinsatzleitung (KEL) (*n* = 72), jeweils relative Angaben zur Anzahl der Antworten in der Untergruppe. Ergänzend findet sich unter der Abbildung eine Tabelle mit den absoluten Zahlen der Umfrage (eigene Darstellung)

Bei der Auswertung der Frequenz von Übungen zeigt sich in Abb. 4, dass allgemeine und nicht näher spezifizierte Übungen in Krankenhäusern zwar prinzipiell durchgeführt werden, die Verteilung jedoch sehr heterogen ist und von ca. ein Drittel der Krankenhäuser, in welchen noch nie eine Übung durchgeführt wurde, über eine einmalige Durchführung in den letzten 5 Jahren (34,7 %) hin zu jährlicher (19,4 %) und sogar halbjährlicher Durchführung (1,4 %) reicht. Überwiegend (67 %) liegt die Häufigkeit von Übungen in den Krankenhäusern der Umfrage jedoch im Bereich „nie“ und „einmalig in den letzten 5 Jahren“.

Im Bereich der Übungen zum Stromausfall zeigt sich eine Häufung im Bereich der jährlichen Übung. Hier üben 43 % der an der Umfrage teilnehmenden Krankenhäuser jährlich zu dieser Thematik.

Weiterführend zeigt sich bei Anlauftests von Notstromaggregaten im Bereich der halbjährlichen Durchführung eine Häufung. Diese Anlauftests wurden in 67 % der Krankenhäuser halbjährlich durchgeführt.

Übungen im Bereich des Ausfalls der Trinkwasserversorgung und Abwasserentsorgung sind sehr selten. Die Mehrzahl der Krankenhäuser hat noch nie eine Übung zu diesem Thema abgehalten. Die Häufigkeit von Übungen im Bereich Trinkwasserversorgung ist dabei etwas höher als im Bereich der Abwasserentsorgung.

**Abb. 5:**
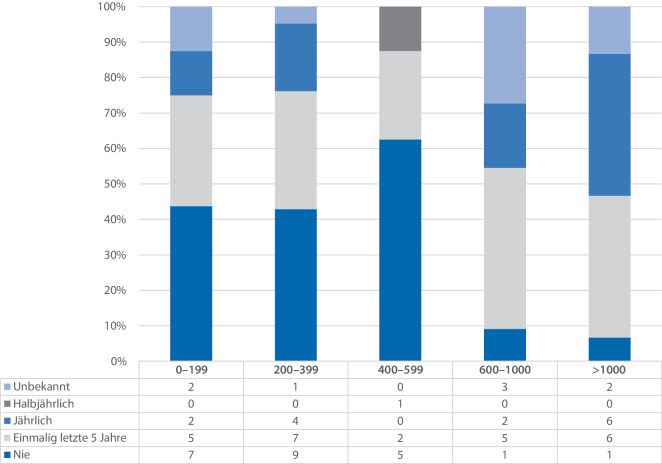
Vergleich der Häufigkeiten von Übungen der Krankenhauseinsatzleitung (KEL) (allgemein, ohne nähere Spezifizierung) in Abhängigkeit von der Bettenzahl (*n* = 71, Ausschluss einer Antwort, die unbekannte Bettenzahl und Übungshäufigkeit angab), jeweils relative Angaben zur Anzahl der Antworten in der Untergruppe. Ergänzend findet sich unter der Abbildung eine Tabelle mit den absoluten Zahlen der Umfrage (eigene Darstellung)

Bei nicht näher spezifizierten Übungen der Krankenhauseinsatzleitung zeigt sich, dass kleinere und mittlere Krankenhäuser (< 599 Betten) seltener oder nie Übungen durchführen im Vergleich zu großen (> 1000 Betten), in welchen die Mehrzahl entweder in den letzten 5 Jahren oder jährlich allgemeine Übungen mit der Krankenhauseinsatzleitung durchführte. Auch unter den Krankenhäusern mit 600 bis 1000 Betten hat die Mehrzahl in den letzten 5 Jahren einmalig eine Übung durchgeführt. Es ist ein Muster erkennbar, dass die Übungsfrequenz mit der Bettenzahl ansteigt und somit mit der Größe des Krankenhauses positiv korreliert. Die Zusammenhänge sind in Abb. 5 grafisch dargestellt.

### Arten von durchgeführten Übungen

**Abb. 6:**
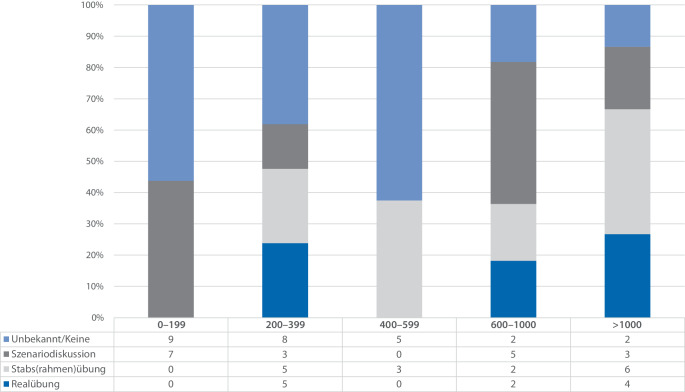
Vergleich der Arten von durchgeführten allgemeinen, nicht näher spezifizierten Übungen der Krankenhauseinsatzleitung (KEL) in Abhängigkeit von der Bettenzahl des befragten Krankenhauses (*n* = 71, Ausschluss einer Antwort, die unbekannte Bettenzahl und Übungshäufigkeit angab, jeweils relative Angaben zur Anzahl der Antworten in der Untergruppe). Ergänzend findet sich unter der Abbildung eine Tabelle mit den absoluten Zahlen der Umfrage (eigene Darstellung)

Neben der Frequenz von Übungen ist auch die Art der durchgeführten Übung entscheidend. Die Aufschlüsselung der Arten von Übungen im Vergleich zur Bettenzahl zeigt mehrere Besonderheiten. Bei allgemeinen Übungen der Krankenhauseinsatzleitung (KEL) ist zu erkennen, dass kleinere Krankenhäuser keine Realübungen und keine Stabsübungen durchführen. Auch die Art der Übung „Unbekannt“ ist vergleichsmäßig groß. Krankenhäuser mit 200 bis 399 Betten sind hier heterogener vertreten, jedoch ist auch hier die Gruppe „Art der Übung Unbekannt“ am größten. Hier zeigt sich weiterführend, ähnlich wie in Abb. 6, dass die Gruppe Art der Übung „Unbekannt“ in großen Krankenhäusern geringer ist als in kleineren Krankenhäusern (< 600 Betten). Zudem ist zu erkennen, dass Stabsübungen in Krankenhäusern mit > 1000 Betten einen hohen Stellenwert einnehmen, gefolgt von Realübungen und Szenariodiskussionen. Zusammenfassend sind 15,5 % der durchgeführten Übungen Realübungen, 22,5 % Stabs(rahmen)übungen, 25,4 % Szenariodiskussionen und 36,6 % unbekannt.

## Diskussion und Bewertung der Ergebnisse

### Limitationen und Übertragbarkeit der Ergebnisse

Die Ergebnisse der Umfrage sind aufgrund der Erhebungsart und Stichprobengröße nur bedingt auf die Krankenhauslandschaft in Deutschland übertragbar. Bei einer Gesamtzahl von 1887 Krankenhäusern in Deutschland (Stand 2021) [14] entsprechen die erhobenen 72 Krankenhäuser etwa 3,8 %. Die Umfrage liefert dennoch einen ersten Überblick zur Fragestellung der Stabsstrukturen in Krankenhäusern, der Intervalle und der Art von Übungen, die sonst noch wenig untersucht werden.

Es ergeben sich weitergehend Einschränkungen in der regionalen Übertragbarkeit der Ergebnisse (s. Abschn. „Gesamtzahlen der Umfrage und statistische Eingrenzungen“). Aus den Bundesländern Niedersachsen und Schleswig-Holstein gibt es keine Teilnahmen an der Umfrage. Die Bundesländer Baden-Württemberg (16), Nordrhein-Westfalen (24) und Rheinland-Pfalz (10) erlauben eine ausreichende Übertragbarkeit.

Die aufgeführten lokalen Einschränkungen lassen sich teilweise durch die Art der Verteilung der Umfrage erklären. Bspw. ist die Umfrage nur von wenigen Landeskrankenhausgesellschaften verteilt worden, darunter zwei der stark vertretenen Bundesländer. Zu Bundeswehrkrankenhäusern sind aufgrund mangelnder Teilnahmen keine Aussagen möglich. Die dargestellten Limitationen zeigen, dass die gewählte Form der Verteilung der Umfrage nicht vollumfassend zweckdienlich war. Direkte Anfragen bei unterschiedlich großen, privaten Krankenhausbetreibern wurden aufgrund von Sicherheitsbedenken abgelehnt. Es erscheint, dass Krankenhäuser in öffentlicher Trägerschaft der Thematik offener gegenüberstehen und daher häufiger an der Umfrage teilgenommen haben.

Zusätzlich ist die Gruppe kleinerer Krankenhäuser stark unterrepräsentiert und die größerer stark überrepräsentiert, wie bereits oben dargestellt.

Die Abweichung kann evtl. durch die Präsenz der Thematik in größeren Krankenhäusern, die zur Verfügung stehenden (Personal‑)Ressourcen und auch die Wichtigkeit dieser Krankenhäuser für die überörtliche Versorgung erklärt werden.

### Diskussion und Bewertung der Ergebnisse zu Stabsstrukturen

Bei einer Analyse der verwendeten Stabsstrukturen ergibt sich trotz Empfehlungen des BBK für die Strukturen der FwDV 100 [12] nur ein Anteil von 47 % an Krankenhäusern, die diese nutzen. Die häufigste Anwendung finden diese Strukturen dabei in Krankenhäusern mit 200 bis 399 Betten sowie mit > 1000 Betten. Wiederum arbeiten kleinere Krankenhäuser mit weniger als 200 Betten häufiger in anderen Strukturen, vorwiegend in den Regelstrukturen oder einer Mischform. Ein Stab nach FwDV 100 benötigt in voller Stärke mindestens 7 bzw. 8 Personen. Ein derartiger Personalaufwand ist v. a. in kleineren Krankenhäusern schwerer zu leisten als in Größeren. Zudem ist der organisatorische Aufwand der Krisenbewältigung in kleineren Krankenhäusern vermutlich geringer als in großen Krankenhäusern (der Maximalversorgung). Eine Erklärung für die häufige Verwendung in Krankenhäusern mit 200 bis 399 Betten ist an dieser Stelle nicht möglich. Es lässt sich allgemein jedoch der Trend erkennen, dass die Strukturen der FwDV 100 in größeren Krankenhäusern häufiger sind und hier eine gewisse Eignung anzunehmen ist, auch wenn einzelne große Krankenhäuser bewusst von diesen Strukturen abweichen, da sie diese als nicht ganz geeignet bewerten. Es lässt sich eine Lücke in der Eignung für kleine Krankenhäuser vermuten, welche zu adressieren ist. Die FwDV 100 beinhaltet neben der vollständigen Besetzung aller Sachgebiete S1–S6 die Möglichkeit, Stäbe nur mit den Sachgebieten S1–S4 zu besetzen oder dies gar über die Zusammenlegung auf 2 bis 3 Personen zu reduzieren.

### Diskussion und Bewertung der Ergebnisse zu Übungen

Ca. ein Drittel der befragten Krankenhäuser hat noch nie eine Allgemeine Übung der KEL durchgeführt. Eine vergleichbare Umfrage in Baden-Württemberg stellt fest, dass 75 % der dortigen Krankenhäuser Übungen zu Gefahren- und Schadenslagen durchführen [7], was einem leicht höheren Anteil entspricht. Die beübten Szenarien wurden nicht erfragt. Betrachtet man die Übungen zum Thema Stromausfall, finden diese häufiger statt als andere Übungsthemen. Es ist denkbar, dass die politische Lage im Winter 2022/2023 mit einer möglichen Gasmangellage und die normativen Verpflichtungen zur Überprüfung von Notstromaggregaten das Thema präsenter und bewusst machen.

Die genannten Anlauftests sind nach DIN 6280-13:1994-12 wie auch DIN VDE 0100-718 monatlich verpflichtend und sollten somit von allen Krankenhäusern regelmäßig durchgeführt werden. Dennoch gaben 11 Krankenhäuser „nie“ an und 4 Krankenhäuser „einmal in 5 Jahren“. In welcher Form die Einhaltung der Norm kontrolliert wird, ist nicht bekannt. Es sollte weiterführend untersucht werden, wie diese Abweichungen zu den normativen Vorgaben zustande kommen.

Ein weiterer relevanter Punkt ist die Frequenz der Übungen zu den Themen Ausfall der Trinkwasserversorgung und Einschränkungen der Abwasserentsorgung. Diese wurden in den befragten Krankenhäusern sehr selten durchgeführt. Hier sind als Gründe einerseits ein fehlendes Bewusstsein denkbar, aber auch die Komplexität der Thematik. Zudem ist eine Realübung wie bei Notstrom mit Schwarzschaltung und Ersatzversorgung nur schwerlich möglich, da die hygienischen Anforderungen an Trinkwasser hoch sind, sodass eine Einspeisung von Wasser in das Krankenhausnetz zu Übungszwecken schwer zu vertreten ist.

Weitergehend zeigt sich, dass die Übungsfrequenzen in kleinen Krankenhäusern geringer sind als in großen Krankenhäusern. Hierbei sind die notwendigen personellen und finanziellen Ressourcen als Einflussfaktor aufzugreifen. Von der Durchführung von Szenariodiskussionen über Stabsübungen bis hin zu Realübungen nimmt der Aufwand stetig zu. Dasselbe gilt für den Aufwand der Vorbereitung. Hierbei ist ein vergleichbarer Aufwand unabhängig von der Größe des Krankenhauses anzunehmen. Daher unterliegen auch hier die kleineren Krankenhäuser den Einschränkungen ihrer verfügbaren Ressourcen. Da Notwendigkeit und Nutzen dieser Übungen erwiesen sind [11, 16], sollte hier über die flächendeckende Einführung einer alternativen Finanzierung durch die zuständigen Ministerien diskutiert werden.

## Fazit für die Praxis

- Jedes Krankenhaus sollte Arbeitsweisen und Arbeitsstrukturen individuell betrachten und daraus bedarfsorientierte und angepasste (Stabs‑)Strukturen für die Bewältigung von Ereignissen ableiten. Hierbei kann von der empfohlenen Stabsstruktur nach FwDV 100 abgewichen werden, was in der Praxis bereits erfolgt.
- Da die Vorbereitung in kleineren Krankenhäusern aufgrund des hohen Ressourcenbedarfs geringer ausgeprägt ist, könnten kleinere Krankenhäuser über die direkte Vernetzung mit größeren Krankenhäusern oder den interdisziplinären Austausch in Fachgesellschaften profitieren.
- Regelmäßige Übungen tragen zur Stärkung der Resilienz bei, erhöhen die Wahrnehmung, und gewonnene Erkenntnisse können in das Risiko- und Krisenmanagement aller beteiligten Akteure einfließen.
- Die Umfrage zeigt, dass die Beübung von Stromausfällen und die Notstromversorgung gut abgedeckt sind. Dies sollte auf die Vorbereitung der Trinkwasserversorgung und Abwasserentsorgung übertragen werden, da hier große Lücken zu erkennen sind.

## Supplementary Information

Fragebogen der Unlineumfrage

## Data Availability

Die Daten können auf Anfrage von den Autoren zur Verfügung gestellt werden.
