# Supplementary material for: Failure of critical infrastructures in German hospitals: Online survey on crisis management structures and exercises in hospitals
Source: Anaesthesiologie. 2026 Mar 27;75(6):391–8. [Article in German] doi: 10.1007/s00101-026-01664-4 (PMC13226423; doi:10.1007/s00101-026-01664-4)
Supplement: Supplementary file 1 — Fragebogen der Unlineumfrage [file 101_2026_1664_MOESM1_ESM.pdf]

Zusatzmaterial zum Beitrag „Ausfall kritischer Infrastrukturen in deutschen Krankenhäusern" von Manuel Geiger, Steffen Neuner, Muhammed Enes Bodur und Alexander Fekete (2026) in Anaesthesiologie.

Beitrag und Zusatzmaterial stehen Ihnen auf www.springermedizin.de zur Verfügung. Bitte geben Sie dort den Beitragstitel in die Suche ein.

Fragebogen der Onlineumfrage

Fragen zum Krankenhaus

- In welchem Bundesland liegt ihr Krankenhaus?
- Wer ist Träger des Krankenhauses? (Single Choice)
    - Bundeswehrkrankenhaus
    - Universitätsklinik
    - Kommunaler Träger
    - Kirchliche Organisation/ Privatwirtschaftliches Unternehmen
    - Sonstige
- Bitte geben Sie den Namen ihres Krankenhauses ein:
    - *(Hinweis: Der Name des Krankenhauses wird nach Auswertung der Umfrage gelöscht. Dieser wird nie veröffentlicht werden oder anderweitig Dritten zugänglich werden und lediglich von den Projektmitarbeitenden von NOWATER in der Auswertung verwendet. Hintergrund der Frage ist, dass diese Umfrage auf unterschiedlichen Wegen verteilt wird und somit mehrfach im selben Krankenhaus, jedoch bei unterschiedlichen Personen ankommen kann. Mit dem Namen des Krankenhauses können wir diese Dopplungen identifizieren, um unsere Auswertung möglichst genau zu gestalten.)*
- Wie viele stationäre und ambulante Betten hat ihr Krankenhaus?
    - (Eingabe Freitext 2x)
- Nimmt ihr Krankenhaus an der Notfallversorgung teil? (Single Choice)
    - Ja
    - Nein
    - Unbekannt
- Ist ihr Krankenhaus ein Krankenhaus der Maximalversorgung? (Single Choice)
    - Ja
    - Nein
    - Unbekannt

Inhaltliche Fragen zu Stabsstrukturen und Stabsarbeit

- Welche Stabsstrukturen werden in Ihrem Krankenhaus in der Krankenhauseinsatzleitung verwendet? (Single Choice)
    - Stab entsprechend Leitfaden KAEP/ FwDV 100
    - Ressortstab
    - Mischform
    - Regelstrukturen
    - Sonstige (Freitext)
- **Befinden sich** in Ihrer Krankenhauseinsatzleitung Verbindungspersonen aus anderen Kritischen Infrastrukturen oder von Feuerwehr, Rettungsdienst oder Polizei? (Single Choice)
    - Ja
    - Nein
    - Unbekannt
- **Entsenden Sie** Verbindungspersonen in andere Stäbe? (Single Choice)
    - Ja
    - Nein
    - Unbekannt

- Wie oft werden in Ihrem Krankenhaus Übungen zum Ausfall von Kritischen Infrastrukturen (Allgemeine Übungen, Strom, Wasser, etc.) durchgeführt? Und in welcher Form werden diese durchgeführt?
  - *Hinweis: An dieser Stelle sind Übungen zum Verhalten im Brandfall und Räumungs-/Evakuierungsübungen nicht mitzuzählen.*
  - *Übungen zum Stromausfall (Frage 2) sind nur dann zu werten, wenn entweder die Krankenhauseinsatzleitung an der Übung beteiligt ist oder ein „echter" Blackout Test gemacht wird und die Lastgänge auf die Notstromversorgung aufgeschaltet werden. Reine „Anlauftests" von Notstromerzeugern sind in Frage 3 aufzuführen.*

- (Abfragematrix)
  - Allgemeine Übungen der Krankenhauseinsatzleitung
  - Nie/ einmalig in den letzten 5 Jahren/ Jährlich/ Halbjährlich
  - Realübung, Stabsrahmenübung, Szenariodiskussion, Sonstige
- Übungen zum Stromausfall (Krankenhauseinsatzleitung/Blackout Test)
  - Nie/ einmalig in den letzten 5 Jahren/ Jährlich/ Halbjährlich
  - Realübung, Stabsrahmenübung, Szenariodiskussion, Sonstige
- Anlauftest bzw. technische Überprüfung von Notstromerzeugern
  - Jährlich/ Halbjährlich/ Quartalsweise/Monatlich
- Übung zum Ausfall der Trinkwasserversorgung
  - Nie/ einmalig in den letzten 5 Jahren/ Jährlich/ Halbjährlich
  - Realübung, Stabsrahmenübung, Szenariodiskussion, Sonstige
- Übungen mit Beeinträchtigung der Abwasserentsorgung
  - Nie/ einmalig in den letzten 5 Jahren/ Jährlich/ Halbjährlich
  - Realübung, Stabsrahmenübung, Szenariodiskussion, Sonstige
